# Mixing of Honeybees with Different Genotypes Affects Individual Worker Behavior and Transcription of Genes in the Neuronal Substrate

**DOI:** 10.1371/journal.pone.0031653

**Published:** 2012-02-14

**Authors:** Tanja Gempe, Silke Stach, Kaspar Bienefeld, Martin Beye

**Affiliations:** 1 Department of Genetics, Heinrich Heine University, Duesseldorf, Germany; 2 Institute for Bee Research, Hohen Neuendorf, Germany; Université Paris 13, France

## Abstract

Division of labor in social insects has made the evolution of collective traits possible that cannot be achieved by individuals alone. Differences in behavioral responses produce variation in engagement in behavioral tasks, which as a consequence, generates a division of labor. We still have little understanding of the genetic components influencing these behaviors, although several candidate genomic regions and genes influencing individual behavior have been identified. Here, we report that mixing of worker honeybees with different genotypes influences the expression of individual worker behaviors and the transcription of genes in the neuronal substrate. These indirect genetic effects arise in a colony because numerous interactions between workers produce interacting phenotypes and genotypes across organisms. We studied hygienic behavior of honeybee workers, which involves the cleaning of diseased brood cells in the colony. We mixed ∼500 newly emerged honeybee workers with genotypes of preferred Low (L) and High (H) hygienic behaviors. The L/H genotypic mixing affected the behavioral engagement of L worker bees in a hygienic task, the cooperation among workers in uncapping single brood cells, and switching between hygienic tasks. We found no evidence that recruiting and task-related stimuli are the primary source of the indirect genetic effects on behavior. We suggested that behavioral responsiveness of L bees was affected by genotypic mixing and found evidence for changes in the brain in terms of 943 differently expressed genes. The functional categories of cell adhesion, cellular component organization, anatomical structure development, protein localization, developmental growth and cell morphogenesis were overrepresented in this set of 943 genes, suggesting that indirect genetic effects can play a role in modulating and modifying the neuronal substrate. Our results suggest that genotypes of social partners affect the behavioral responsiveness and the neuronal substrate of individual workers, indicating a complex genetic architecture underlying the expression of behavior.

## Introduction

The complexity and internal cohesion found within colonies of social insects stem from the coordinated behavioral activities of their colony members. The combined forces of potentially millions of individual workers allow colonies to modify their environments more efficiently, resulting in the tremendous ecological success of social insects in terrestrial ecosystems [1]. In the most advanced insect societies, such as those of honeybees (*Apis mellifera*), there is a strong reproductive division of labor into worker and queen castes. The queen reproduces, while the workers perform tasks mostly related to colony growth and development. Workers can further specialize in a subset of behavioral tasks, resulting in a division of labor among workers [2]–[4]. Coordinated worker behaviors allow for the manifestation of complex, sophisticated traits that would be unachievable by single individuals, such as the building of intricate nest structures, the development of effective collective defense systems against diseases and predators and the ability to locate and exploit ephemeral food sources across the landscape.

How these complex, coordinated worker behaviors are genetically controlled is still not well known [4]–[6]. The coordinated honeybee worker behaviors arise in part because individuals show differences in behavior in response to a given stimulus (behavioral response) that can change with the age of a worker [7], [8], experience [9] and genotype [8], [9]. These differences in behavioral responses to a given stimulus produce variation regarding engagement in behavioral tasks, which as a consequence, generates a division of labor. Younger workers engage in tasks within the nest, such as nursing the brood, whereas older workers perform outside tasks, such as foraging and defense [10]. One genetic component of this age-dependent behavior is the *foraging* (*for*) gene, which encodes a cGMP-dependent protein kinase (PKG). Increased expression of the *for* gene in the brain directs engagement in foraging behavior [11]. Genotypic variation among honeybee workers can explain a substantial portion of the behavioral differences found in a colony [12]–[15]. In the case of pollen foraging, stinging behavior, hygienic behavior and individual response thresholds, the genotypic component of behavior has been mapped to specific genomic regions [16]–[20] harboring candidate genes [21].

Another genotypic effect on behavior arises from the numerous interactions of worker phenotypes in a colony, representing so-called indirect genetic effects [22], [23]. Because many colony-level traits and worker behaviors rely on worker interaction, there is a genotypic component that arises from the phenotypic interaction of workers. As a consequence, genotypic differences in a colony can have strong effects on colony growth, development and fitness as well as individual behavior [24]–[31]. These indirect genetic effects have been repeatedly demonstrated at the level of colony outcomes. When workers from different genotypic sources are combined, the outcome at the level of the colony is often different from what is additively expected from the colony outcomes of the pure genotypes. These indirect effects have been shown to influence thermoregulation, colony growth, colony performance and, in the case of the ant *Solenopsis invicta*, the number of queens in a colony [25]–[28].

The routes through which these indirect genetic effects affect colony outcomes and individual behaviors are not well understood. When honeybees from different genotypic sources are combined into a single colony, the behaviors of single workers can change in response to the genotype of social partners. For instance, when 25% of bees with a genotype of high preference for removing diseased brood from brood cells (hygienic behavior) were mixed with non-hygienic bees in the same colony, the hygienic bees were found to be more persistent at the task compared to individuals from pure hygienic colonies [29]. These behavioral differences have been attributed to indirect genetic effects on task-related stimuli. Workers that engage in a specific task due to their genotype associated behavioral preference will lower the task-related stimuli, and as a consequence, the behavioral response of other workers [26], [29], [30]. Therefore, honeybee workers with genotypes that have high preference for pollen foraging increase the pollen stores in a colony, consequently lowering the engagement of workers that have genotypes with a low pollen foraging preference [30], [31].

Here, we readdress the question of how worker behaviors are genetically influenced by social partners through the simultaneous study of effects on colony-level outcomes, individual behavior, and gene expression in the brain. We examined indirect genetic effects on hygienic behavior [18], [19], [29], [32], which we repeatedly induced in different colonies by mixing newly emerged worker bees with genotypes that have Low (L) and High (H) preferences for hygienic behaviors. We then measured how the indirect genetic effects of genotypic mixing affected the hygienic behavioral activities of single worker bees, collective performance at single treated cells and colony performance. We further examined plausible mechanisms underlying how indirect genetic effects are induced. The behavioral activities can be easily quantified: several workers usually uncap the wax cap of a brood cell that facilitates the final removal of dead pupae by other workers. The number of uncapped brood cells or removed diseased brood at the colony level is a result of the cumulative hygienic activities of single workers. Here, we report that the mixing of genotypes in groups of honeybees affect the behavioral responsiveness and the neuronal substrate of individual workers, suggesting that genotypes of social partners can indirectly affect the expression of individual worker behavior.

## Results

**Figure 1:**
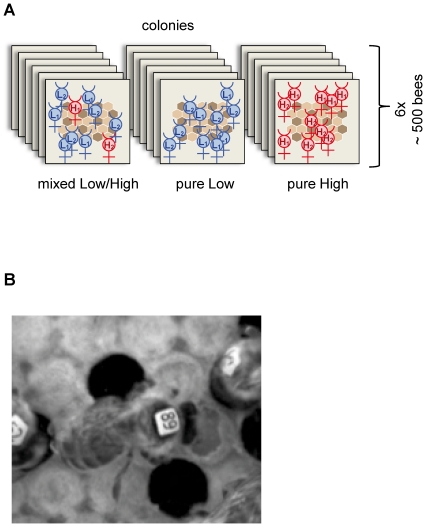
Hygienic behavior of honeybee workers in the Low (L), High (H), and Low/High (L/H) mixed groups. (A) Schematic representation of the experimental setup of the cages. Newly emerged L and H worker bees from the two L (L1 and L2) and two H (H1 and H2) genetic hygienic behavior sources were used to establish mixed L/H, pure L and pure H bee cages. Each cage consisted of ∼500 individually marked bees. L bees are shown in blue, and H bees are shown in red. (B) An example of a worker (tag no. 89) that engaged in uncapping behavior. The hygienic behavior of ∼12-day-old workers was monitored for 12 hours in a brood comb that contained ∼33 pin-killed pupae and control brood cells.

We established two L and two H genotypic worker bee sources based on standard tests of hygienic behavior [29], [33] that measure the uncapping of brood cells and removal of pin-killed brood. We created six L (100% L1+L2), six H (100% H1+H2) and six mixed (80% L1+L2/20% H1+H2) genotypic cages (Figure 1A) by assembling ∼500 newly emerged, individually marked workers from the two L (L1 and L2 in equal proportion) and the two H (H1 and H2, also in equal proportion) genotypic sources for each cage. The groups of bees were placed in small mesh wire boxes, supplied with a comb and food, and maintained in a full-sized queen-right colony. When the experimental worker bees were 12 days old (when worker bees normally perform hygienic behavior at a high frequency), we transferred our ∼500 workers to individual observation combs (Figure 1A). The combs contained ∼33 pin-killed pupae [29], [32], which provided the task-related stimulus to induce hygienic behavior.

**Figure 2:**
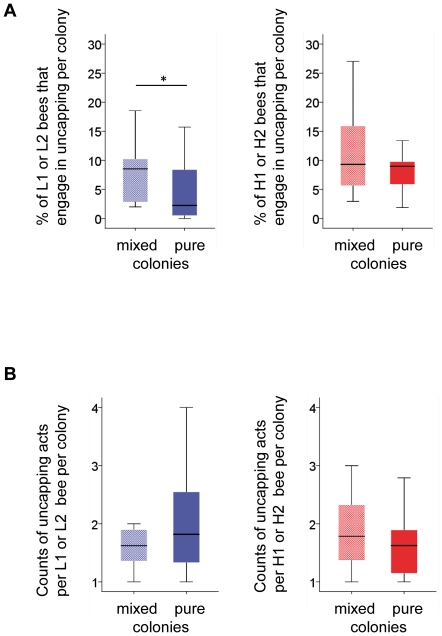
Box plots display the uncapping engagement and performance of L1 and L2 bees (shown in blue) and H1 and H2 bees (shown in red) in the mixed L/H, pure L and pure H bee groups. (A) The proportions (%) of L1 or L2 and H1 and H2 bees that engaged in the uncapping task in the different cages. (B) The counts of uncapping acts per L1 or L2 and H1 and H2 bee that engaged in the uncapping task in the different cages. The differences between L and H bees in the mixed and pure groups in (A) and (B) were compared by *MWU*-tests (* denotes *P*<0.05, *n* = 12).

We monitored the hygienic behavior of ∼9,000 individually marked worker bees. From 6732 individual bees we obtained behavioral information with respect to their hygienic activities (Figure 1B). The proportion of L worker bees that engaged in uncapping behavior increased, approximately doubled, in the mixed L and H bee cages compared to the pure L bee cages (*MWU*-test, *P*<0.05; Figure 2A), suggesting that the presence of H bees increased the frequency of task engagement. However, the number of uncapping acts that an individual L bee performed was not affected by the presence of H workers. The uncapping acts per L worker bee did not differ between the different mixed L/H and pure L groups (*MWU*-test, *P*>0.4; Figure 2B). We aggregated the uncapping data for L bees from the mixed groups and compared the distributions of uncapping acts per L bee to those of pure L groups. The distributions obtained from mixed and pure cages were consistent with a Poisson distribution (*KS*-test, *P*>0.05; Figure S1) if we excluded a single bee that exhibited exceptional performance (>9 uncapping acts) from each dataset. This result suggests that, except for a single bee, the 164 bees that specialized in the uncapping task showed no overrepresented task performance. We then examined whether the switching of L bees between uncapping and removing tasks was affected by the presence of H bees. We again combined data from mixed and pure L cages because of the small sample size. The number of L bees that engaged in uncapping behavior, but not in removing behavior was increased in mixed cages compared to bees from pure L cages (*χ^2^*-test, *P*<0.025, Table S1), suggesting a decreased switching to the other hygienic task (removal). Together, the results showed that the frequency of L bee engagement in uncapping behavior increased, and that switching between hygienic tasks decreased in response to the presence of H workers in the group.

**Figure 3:**
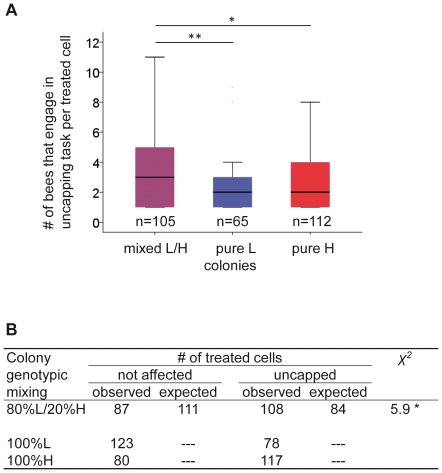
Group behavioral uncapping responses in the mixed L/H and pure L and H bee cages. (A) The number of workers that engaged in uncapping tasks per treated brood cell (boxplot; combined data from different cages). Numbers represent cumulative data from all cages. Medians were compared with the *Median*-test; * denotes *P*<0.05, and ** denotes *P*<0.005. (B) The number of uncapped and non-uncapped cells in a colony as a measure of colony-level performance. The expected value in mixed cages was calculated based on the additive combination of the pure L and H bee colony outcomes and the relative contributions of bees in the mixed cages. * denotes *P*<0.02. We identified 105 treated brood cells at which bees engaged in uncapping behavior, which were analyzed in (A), and 108 treated brood that were actually uncapped in the different genotypic mixed groups.

The mixing of L and H bees also affected collective uncapping behaviors at the single brood cell and at the level of the group outcome. We first analyzed how mixing of genotypes influenced the average number of bees that engaged in the uncapping task at a single treated brood cell. We documented 105 treated brood cells at which bees engaged in uncapping behaviors in mixed cages, 65 treated brood cells in pure L cages and 112 treated brood cells in pure H bee cages (combined data from the different cages). In mixed L/H bee cages, 3 bees (median) engaged in uncapping behavior, compared to 2 bees in L pure and H pure bee cages. The median of 3 bees in the mixed group significantly differed from the median of 2 in both the pure L and pure H bee cages (*median*-test, *P*<0.05, Figure 3A), suggesting that more bees engaged in uncapping single cells in the mixed cages. We next analyzed how the differences in behavior in a mixed cage may affect the group-level outcome. We found that more cells were uncapped in genotypically mixed L/H cages than were expected from extrapolations of the pure group measurements. We compared the numbers of uncapped cells in mixed cages to the expected numbers we calculated from the observed numbers found in pure L and H cages, and the relative proportions of L and H bees introduced into the mixed cages. We found significant differences in the number of uncapped cells between what was additively expected and what was observed for each of the mixed cages or for the combined outcome of all cages (*χ^2^*-test, *P*<0.02; Figure 3B, Table S2). This non-additive improvement in the uncapping of cells links H bee-induced behavioral changes in L bees with the phenotypic outcome at the group level.

In contrast to what was observed for L bees, the uncapping performance of H bees was not affected by the presence of L bees. This is possibly explained by smaller sample size of H bees compared to L bees in genotypic mixed groups and the smaller statistical power. The proportion of H bees engaged in the uncapping task and the number of uncapping acts per H bee did not differ between mixed L/H and pure H cages (Figure 2A, B, *P*>0.4). We also studied the removing behavior of L and H bees. However, the removal of brood from treated cells was not completed after 12 hours of observation. Within the observation time, we detected no differences in the engagement or performance of the bees in the removing task between mixed and pure L or H cages (Figure S2A, B, *P*>0.2).

We next investigated plausible routes through which indirect genetic effects might have influenced L bee uncapping behaviors. Our experimental setup involved mixing the 2 L and 2 H colony sources in cages to show that the mixing itself did not cause the behavioral differences. We conclude that the induced behavioral differences must be due to genotypic differences underlying the L and H phenotypes. We further studied how indirect genetic effects influence behaviors in L bees. Increased uncapping activities of H bees can directly affect the uncapping behaviors of L bees. This is either achieved through H bee uncapping activities that alter the uncapping stimulus for L bees [34] or by evoked social learning in L bees [35]. Previous work has indicated that indirect genetic effects influence task-related stimuli. The effects of the preferred behaviors of one genotypic source can alter task-related stimuli and thus behavioral response of other bees [26], [30], [31]. In the case of uncapping behavior, the initial uncapping of a cell would generate a stronger stimulus, so that more bees respond by exhibiting uncapping behavior at that specific cell. To find general evidence for such recruiting system we first analyzed data from the pure L bee cages. The uncapping acts of L bees (combined data set of all cages) are not randomly distributed across cells. We observed a mean number of 17 uncapping acts per cage that cluster among 7 out of 33 treated brood cells, which differs significantly from a random expectation (*P*<10^−3^). This result indicates that uncapping acts of some bees recruited other bees to uncapping behavior at specific cells.

A plausible hypothesis of L bees increased uncapping engagement in the presence of H bees is that H bees have recruited, by their higher uncapping acts, the L bees to uncapping behavior. To test the hypothesis, we studied the association of H and L bee uncapping acts at the 105 single brood cells in the mixed cages (combined dataset). The null hypothesis, the random association of the H and L bee uncapping acts among the 105 single brood cells, was calculated by applying a binomial distribution and the probability given by the relative proportions of L and H uncapping acts we observed in the mixed cages. We found that the number of L and H bee uncapping acts upon single cells did not deviate from a random association (Table 1, *χ^2^*-test, two-tailed test, *P* = 0.12) The power analysis showed that we should detect moderate to high effects given the sample size (*power* = 0.99, effective size of *ω* = 0.5, and *df* = 4). Indeed, the acts were distributed opposed to the prediction of a simple recruiting and stimulus-based model. For example, we observed 43 cells at which solely L bees showed uncapping behavior, but expected only 31 such cells. We observed 36 cells at which a single H bee together with L bees showed uncapping behaviors, but we expected to find 45 such cells. Thus, our results provide no evidence that H bees have directly recruited L bees to uncapping behavior by a mechanism such as stronger task-related stimuli that arise from the more uncapped cells from H bees. If not recruiting and task-related stimuli are the primary source of the behavioral effects we suggest that behavioral responsiveness of L bees was affected by genotypic mixing. Changes in responsiveness may also explain the array of effects on uncapping behavior including effects on uncapping engagements, switching between tasks and cooperation at single brood cells.

**Table 1:** The distribution of H bee and L bee uncapping activities among the 105 uncapped brood cells in mixed L/H bee groups.

# of H bee acts per cell	Expected # of cells	Observed # of cells	*χ^2^*
0	31	43	
1	45	36	
2	23	19	
3	5	5	
4	1	1	
≥5	–	1	
			8.7

We used the relative proportion of H to L bee acts in the mixed groups ( = 0.26) as an estimate of the probability of H bees performing an uncapping activity relative to L bee uncapping activity among the 105 uncapped cells. We used the binomial distribution to estimate the expected number of cells at which 0, 1, 2, 3 or 4 uncapping acts would be expected from H bees in relation to L bee acts. On average, four bees in the mixed groups engaged in the uncapping task at a single cell. The observed and expected numbers of cells in the different categories were compared with a *χ^2^*-test (*df* = 4; *P* = 0.12). Power for this test is 0.99 for ω = 0.5.

Although there are multiple ways how indirect genetic effects can influence the responsiveness of workers (including through pheromones, behavioral interactions or a shared social environment), we note that all those signals should converge at the neuronal substrate, which controls complex behaviors. We next studied genome-wide transcription differences in L worker brains to identify associations of indirect genetic effects on the behavioral phenotype. We studied transcriptional differences in brains from L workers that engaged in uncapping tasks and those that showed no hygienic behavior, originating from mixed L/H and pure L bee cages. We analyzed 132 two-color microarrays (Figure S3) and the transcription profiles of 13,440 genes. We analyzed gene expression with linear models for microarray data (LIMMA) [36] to study the transcriptional correlates of behavior, genotypic mixing, and the combined condition of behavior and genotypic mixing. The expression levels of 943 genes (7% of genes studied) changed significantly in the combined condition (behavior×genotypic mixing, Table 2), suggesting that these differences in L bees are specifically associated with behavioral changes induced by the presence of H bees. We identified 650 genes (5%) that exhibited altered expression levels due to the condition of behavior and 426 genes (3%) with altered expression levels due to the condition of mixing L and H bees (Table 2). We confirmed differential transcription of the *GB16850* and *GB19709* genes (putative orthologs: Tyrosine kinase-like orphan receptor and Synaptotagmin 7, respectively) in L bees performing uncapping in the mixed genotype group compared to L control bees (L bees from the mixed group that did not perform uncapping behavior and L bees from pure groups performing uncapping behavior) by qRT-PCR (*P*<0.05).

**Table 2:** Differences in brain gene expression associated with the conditions of uncapping behavior (denoted as B) and mixing of L/H bees (genotypic mixing denoted as C) and their interaction (BxC) in 76 L strain worker bees.

*P*		B (%)	C (%)	BxC (%)
<0.025	↑	362 (2.7)	250 (1.9)	370 (2.8)
	↓	288 (2.1)	176 (1.3)	573 (4.3)
	Σ	650 (4.8)	426 (3.2)	943 (7.0)

The numbers of up (↑) and down (↓) regulated genes are shown. Comparisons are relative to non-active L bees from pure L bee cages. *P*<0.025 was adjusted for a false discovery rate (FDR) of 5%. The relative proportions (%) were estimated from the 13,440 genes tested. The effects on gene expression partially overlapped. Of the 943 genes associated with BxC conditions, 168 genes were also found by the condition of B and 178 genes by the condition of C. The candidate genes associated with B, C, and BxC are listed in the Tables S3, S4, S5, respectively.

We further studied the functional relationships of the 943 genes that exhibited altered expression in the combined condition of behavior and genotypic mixing. The regulation of these genes is specifically associated with the effect of genotypic mixing and the resulting behavior. We used these genes as candidates to gain insights into the underlying biological processes. We functionally annotated and grouped genes by similar molecular processes and biological functions by assigning GO terms in the FatiGO and DAVID databases. The biological processes of cell adhesion, cellular component organization, anatomical structure development, protein localization, developmental growth and cell morphogenesis were significantly overrepresented. The overrepresentation of these functional categories suggests that neuronal cellular function associates with the uncapping differences that were influenced by indirect genetic effects.

We next grouped the genes into co-regulated modules [37] to further delineate their functional relationships. This approach has been successful in unraveling gene networks affecting complex traits [38]. We computed the pairwise correlations of the transcriptional levels of the 943 candidate genes. To perform the analysis, we utilized the log-transformed data from our two-color arrays and further examined the expression differences between bees performing uncapping behavior in genotypically mixed and pure groups. We identified 11 modules consisting of 8 to 268 (median = 28 genes) strongly co-regulated genes (Table 3 and Table S6). Ten of the 11 modules were comprised of sets of co-regulated genes that were unique to the combined condition of behavior and genotypic mixing. These modules had, on average, 53% unique sets of co-regulated gene pairs (Table 3) that were not detected in the analyses of the single condition of either behavior or genotypic mixing. Thus, genotypic mixing regulates defined sets of co-regulated gene networks in the brains of workers that associate with the behavioral effects. We further examined whether we could assign specific molecular processes and biological functions to the genes that we grouped into co-regulated transcriptional modules (Table S6). For none of the single modules we were able to detect overrepresentation of GO terms, possibly because gene number in most modules were low. We next studied biological processes that were repeatedly found across the different co-regulated modules. We identified in 8 modules sets of genes (representing 17–39% of orthologs that we found in the modules) that are implicated in the regulation of the processes of differentiation (Figure S4A, B and Tables 3, S6) including genes associated with cell to cell signaling and transcriptional regulation (Tables 3, S6). In six modules, we repeatedly found sets of genes that are involved in controlling the connectivity of the neuronal substrate (representing 11–33% of orthologs that we found in the modules), including genes involved in neurotransmitter metabolism, ion transport and transmission of nerve impulses (Figure S4A–C and Tables 3, S6). Together, the lists of genes from our modules and overrepresentation analyses suggest that indirect genetic effects on behavior are associated with gene expression changes that may modulate and modify the neuronal substrate.

**Table 3:** Modules of co-regulated transcripts identified among the 943 candidate genes that exhibited altered expression in the combined condition of behavior and genotypic mixing.

Module ID	|*r^—^*|	# of transcripts	Proportion of pairwise correlations			% of ortho-logs identi-fied in *D. melanogaster*	% of orthologs assigned	
				not identified by the effects of			with the functional process	
				B	C		Differentiation	Neuronal connectivity
M1	0.81	21	100	0	7.1	4.8	–	–
M2	0.66	35	97.0	12.5	54.7	17.1	0	33.3
M3	0.63	12	93.9	80.6	87.1	66.7	37.5	12.5
M4	0.62	28	98.9	20.6	34.2	64.3	38.9	22.2
M5	0.56	268	>85	>19	>48	60.8	28.8	6.1
M6	0.54	21	98.6	77.3	56.0	85.7	16.7	0
M7	0.46	6	93.3	71.4	85.7	50.0	–	–
M8	0.32	36	67.9	27.3	64.7	72.2	23.1	11.5
M9	0.30	21	76.2	60.0	66.3	76.2	37.5	0
M10	0.33	49	95.8	46.3	53.2	91.8	28.9	0
M11	0.28	79	87.2	34.5	49.7	67.1	24.5	15.1

|*r^—^*| denotes the average correlation as estimated from the mean of all pairwise correlations of transcripts within a module. B denotes the condition of behavior, and C represents the condition of genotypic mixing. Relative proportions were calculated from the entire set of pairwise correlations within the modules. The percentage of identified orthologs in *Drosophila melanogaster* was obtained from the UIUC Honey Bee oligo 13K v1 annotation file (May 2007).

## Discussion

Our results showed that genotypic mixing can modify gene expression in the neuronal substrate and can influence the uncapping behaviors of worker bees, the latter affecting the group performance. We showed that this indirect genetic effect has a variety of implications on uncapping behavior including effects on the uncapping engagement, the cooperation of workers at single cells, and the switching between hygienic tasks.

**Figure 4:**
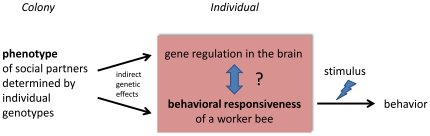
Model of the indirect genetic effects on behavioral responsiveness and gene expression in the brain.

The distribution of H and L bee acts across single cells provided evidence that the indirect effects on behavior do not rely primarily on a simple recruiting mechanism in which H bees have directly recruited L bees into uncapping behavior that includes a stimulus based mechanism arising from the higher number of uncapped cells. We conclude that genotypic mixing has affected the internal condition that primarily influenced the behavioral response to a given stimulus (the behavioral responsiveness) (Figure 4). We found evidence for such changes in the brain in terms of 943 genes that were differently expressed that associated with indirect genetic effects on behavior. We detected that the functional categories of cell adhesion, cellular component organization, anatomical structure development, protein localization, developmental growth and cell morphogenesis were overrepresented in this set of 943 genes, suggesting that indirect genetic effects can play a role in modulating and modifying the neuronal substrate. However, we don't know whether these changes in the neuronal substrate caused the behavioral effects, or whether these changes are the product of altered behaviors (Figure 4). In addition, our study showed that indirect genetic effects also changed the group-level outcome by adjusting the behavioral performance and cooperation of individual workers.

Indirect genetic effects [23], including those related to hygienic behaviors and pollen foraging behaviors of individual worker bees, have been repeatedly observed in social insects [29], [30], [39]. The indirect genetic effects are thus far thought to be mediated via a task-related stimulus. The preferred behavioral task engagements of workers of a given genotype will affect task-associated stimuli and as a consequence the behavioral responses of other workers [7], [26], [30], [31], [40]. This model is consistent with several effects observed in genotypically mixed colonies, including behavioral effects on pollen hoarding, hygienic behavior and thermoregulation [26], [29], [30]. For instance, in colonies in which bees with high (H) and low (L) pollen-hoarding genotypes are combined, the number of L bees hoarding for pollen is decreased compared to colonies consisting solely of bees of the L pollen-hoarding genotype. H workers pollen-foraging activities increase the pollen stores in a colony, thus reducing the pollen-hoarding stimulus to engage others, particularly L bees, in the pollen-hoarding task [30], [31]. Our findings offer an extension to the stimulus-based model in that the behavioral responsiveness of worker bees can also be modulated by indirect genetic effects. This finding suggests that the individual genotypes together with the genotypes that residue in social partners influence the expression of individual uncapping behavior.

Our study followed the experimental setup of Arathi and Spivak [29] in which hygienic and non-hygienic bees were mixed. They reported that the proportion of non-hygienic bees performing hygienic behaviors in mixed colonies decreased, whereas it increased in this study, although the proportion of hygienic and non-hygienic bees in mixed colonies was about the same across the studies. A possible explanation for this is that our low hygienic bees performed hygienic behavior, whereas in the Arathi and Spivak study, the non-hygienic bees were derived from a low selection line displaying no hygienic behavior. This may also explain other differences observed, such as decreased switching from uncapping to removing in mixed colonies (i.e., increased task partitioning) and non-additive improvement of uncapping at the level of the group, which were not observed in the previous study. Moreover, these indirect genetic effects may be very plastic and change according to the specific genotypes that are combined.

Indirect genetic effects have mostly been studied at the colony level, with colony-level outcomes often differing from what was additively expected from the group outcomes associated with pure genotypes. The outcome from indirect genetic effects in honeybees can be very severe, including those related to colony performance and thermoregulation traits [25]–[28]. An intriguing example is queen numbers in the fire ant *Solenopsis invicta* [41]. A certain proportion of workers with the Bb Gp-9 genotype in a *S. invicta* colony can facilitate changes in social behavior and determine the occurrence of single versus multiple queens in the colony. Our result presented in this study demonstrates that indirect genetic effects on group performance is a direct product of individual worker behavioral changes. We showed that genotypic mixing increased the engagement of L workers in uncapping behavior, reduced switching to another hygienic task and increased the number of cooperating bees at single brood cells.

There are different plausible routes through which indirect genetic effects can be mediated between social partners, including pheromones, direct behavioral contact or a shared social environment. A plausible route how indirect genetic effects can influence gene expression changes in the neuronal substrate is through behavioral experiences. Previous studies have repeatedly shown that neuronal development is not complete when honeybees emerge [42], and is influenced by environmental conditions. Microglomerular structure and density are developmentally reorganized in workers by foraging experience [43], [44]. There is also evidence that neuronal reorganization can induce behavioral changes [45], [46]. Differences in behavioral experience in genotypic mixed and pure groups may influence the behaviors of L bees under subsequent conditions.

Previous studies have shown that genotypic mixing can affect the expression of genes [28], [47], but how this relates to behavior is thus far unknown. We extended this approach in our study to define sets of candidate genes and molecular functions that are specifically associated with the indirect genetic effects on behavior.

Our results indicate that indirect genetic effects are another component of the genetic architecture that shapes the behavioral responsiveness of a worker bee. Other studies in honeybees have repeatedly demonstrated that the behavioral responses of a worker to a given stimulus can change with the age of a worker [7], [8], experience [9], pheromones [48]–[50] and genotype [8], [9]. The genetic variation among honeybee workers can explain a substantial portion of the behavioral differences found among workers in a colony [12]–[16], [18], [20], [51]–[54] and is thought to play a role in the division of labor [7], [40]. Our findings provide evidence that worker behavioral responsiveness is also influenced by the genotype of social partners.

The indirect genetic effects on behavioral responsiveness observed in this study are possibly only part of an indirect genetic component shaping worker behaviors that we are unable to observe in the absence of genetic variation. Indirect genetic components that arise from interactions of worker phenotypes may aid in the understanding how complex innate behaviors of worker bees are orchestrated by just ∼15,000 genes.

## Materials and Methods

### Sources of honeybees

We established two H and two L genetic sources of honeybees (*Apis mellifera*), which showed high and low hygienic performance, respectively, in standard tests of hygienic behavior that measure the number of removed pin-killed pupae from sealed brood cells [29]. We pooled semen from 8 drones that were derived from single mother queens. We utilized two semen pools to inseminate 2 queens, each derived from different colonies of a selection line for high hygienic behavior to establish 2 H source colonies. The selection line was established through 10 generations of selection for the hygienic behavioral performance of worker bees [33]. We used semen pools to inseminate 2 queens, each derived from different unrelated L colonies that were identified using pedigree information in population screens (www.beebreed.eu). We confirmed the high and low hygienic performance of our two H (H_1_, H_2_) and L (L_1_, L_2_) source colonies using the standard test of hygienic behavior based on the amount of removed pin-killed brood [29].

### Behavioral assay

We individually marked newly emerged worker bees from H and L genetic source colonies with small colored and numbered tags (Opalith-Plättchen). We combined ∼500 worker bees (a mix from the different donor colonies, of the same age) to establish six pure H bee (100%H = H_1_+H_2_), six pure L bee (100%L = L_1_+L_2_) and six mixed L/H (20%H = H_1_+H_2_ and 80% L = L_1_+L_2_) cages. The bees in each group were maintained separately in mesh wire boxes supplied with a comb, honey and pollen supply in the same foster colony to provide the pheromonal bouquet of a natural colony. Worker bees were analyzed when they were 12 days old, which is the period of time during which they engage in hygienic behaviors at the highest frequency [55]. The bees of each cage were transferred to an observation hive together with a comb that contained brood, honey and pollen. The observation hives were again separated via mesh wire from the full-sized colony. Brood combs for the hygienic behavioral assay were derived from different colonies that were randomly chosen. Within the observation area (∼10×10 cm), approximately 33 pupae in sealed brood cells were pin-killed, and approximately 70 pupae in sealed brood cells were used as non-treated controls. Hygienic behavior was documented for 12 hours under infrared light conditions (LEDs: OSA Opto-Light GmbH, Germany, Type: OIS 330 880) using an infrared-sensitive camera (Panasonic WV-NP1004 megapixel color network IP). The numbers of uncapped cells and removed brood from the pin-treated brood cells greatly exceeded the number of uncapped cells and removed brood for the non-treated cells (*χ^2^*-test, P<0.0001) in our behavioral assays. Although we cannot exclude the possibility that some control cells contained diseased brood, this result showed that hygienic behavior was specifically induced by pin treatments in our behavioral assays. We recorded worker hygienic behavior whether they engaged in the uncapping task (uncapping of sealed brood cells) or the removing task (removal of pupae or parts of them from the brood cell). We recorded the uncapping and removing acts of each individual bee and identified the individual brood cells at which the behaviors were performed in each colony. Only acts of single bees at different cells (treated or non-treated cells) were considered. Bees were categorized as non-hygienic when they did not engage in hygienic tasks and when they were observed at least once in the observation area and exposed to the hygienic stimulus. After the observation period of 12 hours, the bees were shock-frozen and stored at −70°C. We determined the number of H_1_, H_2_, L_1_ and L_2_ bees in each of the 18 cages. We applied the non-parametric Mann-Whitney U-test (*MWU*-test), *Median*- or *χ^2^*-test to compare differences in hygienic behaviors and the Kolmogorov-Smirnov test (*KS*-test) to determine deviations from the Poisson distribution. Statistical analyses were performed using PASW Statistics 18 (SPSS Inc., Illinois) software. Analysis of power was performed using the pwr package in R package software suite.

### Microarray analysis

The honeybee whole-genome oligonucleotide microarray (Design: UIUC Honey bee oligo 13K v1, Accession: A-MEXP-755) contains 28,800 oligos representing 13,440 genes derived from annotations of the honeybee genome sequence [56]. A total of 132 microarrays were used for the profiling of 76 individual brains in a loop design (Figure S3) to analyze the effects of the conditions of uncapping behavior, genotypic mixing and their interaction on gene transcription. Four combinations of behavior and genotypic mixing based on colony type were evaluated for the L strain bees: uncapping L bees from L/H mixed cages, uncapping L bees from pure L cages, non-hygienic L bees from mixed L/H cages, and non-hygienic L bees from pure L cages (uncapping mixed vs. uncapping pure: 48 hybridizations; uncapping mixed vs. non-active mixed: 28 hybridizations; non-active pure vs. non-active mixed: 28 hybridizations; uncapping pure vs. non-active pure: 28 hybridizations). We increased the uncapping pure and uncapping mixed samples to achieve greater statistical power in the subsequent Modulated Modularity Clustering (MMC) analysis. We randomly chose bees from each category and experimental replicate. To improve the statistical power of the subsequent MMC analysis (see below), we increased the number of comparisons between bees from the uncapping pure and uncapping mixed categories. The balanced hybridization design with equal numbers of comparisons produced very similar gene expression results. Total RNA was isolated from individual brains (including the the optical lobes, excluding the retina) using a standard TRIzol protocol with subsequent purification by filter columns (Qiagen, Germany) and column removal of DNA by DNase digestion. A total of 1 µg of RNA was amplified and labeled with mono-reactive cy3 and cy5 fluorophore dyes (Amersham, GE Healthcare) following the protocol of the supplier (MessageAmp II aRNA Amplification kit, Ambion). We hybridized 3 µg of each labeled RNA to a single microarray slide. The slides were scanned (Axon 4000B Scanner), and raw hybridization signals were extracted (GenePix Pro 6.0 software, Agilent Technologies). Transcription-level data were processed and analyzed using the LIMMA 2.16 software package [36], [57]. The quality of hybridization was evaluated using the raw expression data from control probes spotted on each slide. Transcription-level data were treated for background correction (“normexp” function) [58] and intensity-dependent bias detection (“normalizeWithinArrays” function with the default print-tip loess normalization method) [59], and the log-transformed expression ratios were calculated. Data from duplicate spots were averaged using the “avedups” function. We utilized a design matrix that incorporated the conditions of behavior (uncapping behavior, no hygienic behavior) and genotypic mixing (L/H mixed, L pure), and applied the linear models using the Bayesian fitting option. All microarray data are MIAME-compliant, and the raw data have been deposited in a MIAME-compliant database (ArrayExpress, EMBL-EBI) with the accession number E-MTAB-801. Gene transcription differences resulting from the conditions of behavior and genotypic mixing, as well as their combined conditions, were specified as contrasts using linear models. *P* values were adjusted for multiple testing with a 5% false discovery rate (FDR). We applied Modulated Modularity Clustering (MMC) analysis (http://mmc.gnets.ncsu.edu/) to our expression data for 943 candidate genes and uncapping pure/mixed comparisons (*n* = 48). We used pairwise Pearson correlations between the measurements of each transcript to identify modules of highly co-regulated transcripts. Modules consisting of fewer than 5 genes or transcripts exhibiting low connectivity (*r*<0.25) were excluded. We estimated the relative proportion (%) of pairs of correlated transcripts (*P*<0.05) that were not detected in the analysis of the single conditions of either behavior or genotypic mixing. The strength of the co-regulation of transcripts within a module (as estimated from pairwise correlations from 48 comparisons) was visualized using a non-metric multi-dimensional scaling procedure (default setting, PRIMER 6.1.6 software, PRIMER-E). We used the calculated stress value as an estimate of variance. Functional annotation of gene sets that fell into similar categories of GO terms for molecular processes and biological functions were identified using the DAVID database (http://david.abcc.ncifcrf.gov/) [60], [61], and the FatiGO database (http://fatigo.org), which includes an overrepresentation analysis of GO terms. We utilized the gene annotations from the UIUC Honey Bee oligo 13K v1 annotation file (May 2007).

## Supporting Information

Figure S1 **The number of uncapping acts per L bee in mixed L/H and pure L bee cages.** Distributions were compared with a Poisson process. If we exclude a single bee that showed exceptional performance (>9 uncapping acts) from each dataset (combined data from all cages), the distributions are consistent with a Poisson distribution (*KS*-test, *P*>0.05; L bees in mixed colony λ = 0.72; L bees in pure L groups λ = 0.74).
(TIFF)

Figure S2 **Box plots displaying the engagement and performance of removing activities by L bees (shown in blue boxes) and H bees (shown in red boxes) in mixed L/H and pure L or H bee groups.** (A) The proportion (%) of L1/L2 or H1/H2 bees that engaged in removing tasks in the different cages. (B) The counts of removing acts per L1 or L2 and H1 or H2 worker bee in the different cages. Differences between mixed and pure cages were compared by *MWU*-tests (for (A) and (B): *P*>0.2; *n* = 12).
(TIFF)

Figure S3 **Microarray experimental design.** Each box indicates an RNA sample from a bee that exhibited uncapping behavior (square boxes) or a control bee that showed no hygienic behavior (oval boxes). Light blue and dark blue colored boxes were assigned to bees depending on whether they were derived from the mixed L/H bee colony or the pure L bee colony, respectively. Arrows denote the comparison performed on a microarray slide, with the arrow tail being assigned to the Cy3-labeled RNA probe, and the arrowhead being assigned to the Cy5-labeled RNA probe.
(TIFF)

Figure S4 **A network view of three highly co-regulated transcript sets after module formation.** Correlated transcripts are shown in ovals with their gene names. The distances between genes in the plot correspond to their relative connectivity within the network, as calculated by a multiple scaling procedure. |*r^—^*| denotes the average correlation and connectivity of the entire transcript set. GO functional assignment: genes regulating developmental processes are shown in green, and genes controlling neuronal connectivity processes are marked in orange. (A) Module M3 consists of 12 transcripts (stress 0.06). (B) Module M4 includes 28 transcripts (stress 0.21). (C) Module M2 is comprised of 35 transcripts (stress 0.19).
(TIFF)

Table S1 **The number of L bees that engaged in the uncapping task, but not also in the second hygienic task, removing, in mixed L/H and pure L bee groups (cumulated data).** The observed numbers in mixed and pure L groups were compared with a *χ^2^*-test (*df* = 1; *P<0.025).
(PDF)

Table S2 **The number of uncapped brood cells in mixed L/H bee groups (cumulative data from all cages).** The expected numbers of uncapped cells (those that were treated) was estimated from the mean relative proportion of uncapped cells in pure L or H bee groups and the relative proportion of L and H bees in the mixed groups. The mean percentage of uncapped brood cells in H bee groups was 59% and 39% in L bee groups. The observed and expected numbers of uncapped cells in the different cages were compared with a *χ^2^*-test (*df* = 5; **P<0.0005).
(PDF)

Table S3 **Candidate genes associated with uncapping behavior (B).** A modified t-test was performed on the log-transformed expression ratios gained from 132 hybridizations between 76 L strain worker bees. Expression levels relate to non-active L bees from pure L bee colonies. Oligo Ids, Gene Ids, and Comments are obtained from the UIUC Honey bee oligo 13K v1 annotation file (May 2007).
(XLS)

Table S4 **Candidate genes associated with genotypic mixing (C).** A modified t-test was performed on the log-transformed expression ratios gained from 132 hybridizations between 76 L strain worker bees. Expression levels relate to non-active L bees from pure L bee colonies. Oligo Ids, Gene Ids, and Comments are obtained from the UIUC Honey bee oligo 13K v1 annotation file (May 2007).
(XLS)

Table S5 **Candidate genes associated with the interaction (BxC) of uncapping behavior (B) and genotypic mixing (C).** A modified t-test was performed on the log-transformed expression ratios gained from 132 hybridizations between 76 L strain worker bees. Expression levels relate to non-active L bees from pure L bee colonies. Oligo Ids, Gene Ids, and Comments are obtained from the UIUC Honey bee oligo 13K v1 annotation file (May 2007).
(XLS)

Table S6 **The list of genes identified in the modules of highly correlated transcripts and their functions in** ***Drosophila melanogaster*** **orthologs.** Modules of transcriptionally correlated genes inferred from 943 transcripts associated with the interaction of uncapping behavior and hygienic genotype composition (BxC). Pearson correlation analysis was performed on the normalized expression ratios of C+BxC hybridizations (n = 48). |*r^—^*| is the average correlation of all transcripts within a module, *r* the mean correlation of one transcript to the other transcripts in the module. FlyBase IDs represent putative orthologs based on the gene annotations from the UIUC Honey bee oligo 13K v1 annotation file (May 2007). GO terms were assigned using DAVID functional annotation clustering tool.
(XLS)
