# Supplementary figures and images for: Mixing of Honeybees with Different Genotypes Affects Individual Worker Behavior and Transcription of Genes in the Neuronal Substrate
Source: PLoS One. 2012 Feb 14;7(2):e31653. doi: 10.1371/journal.pone.0031653 (PMC3279409; doi:10.1371/journal.pone.0031653)

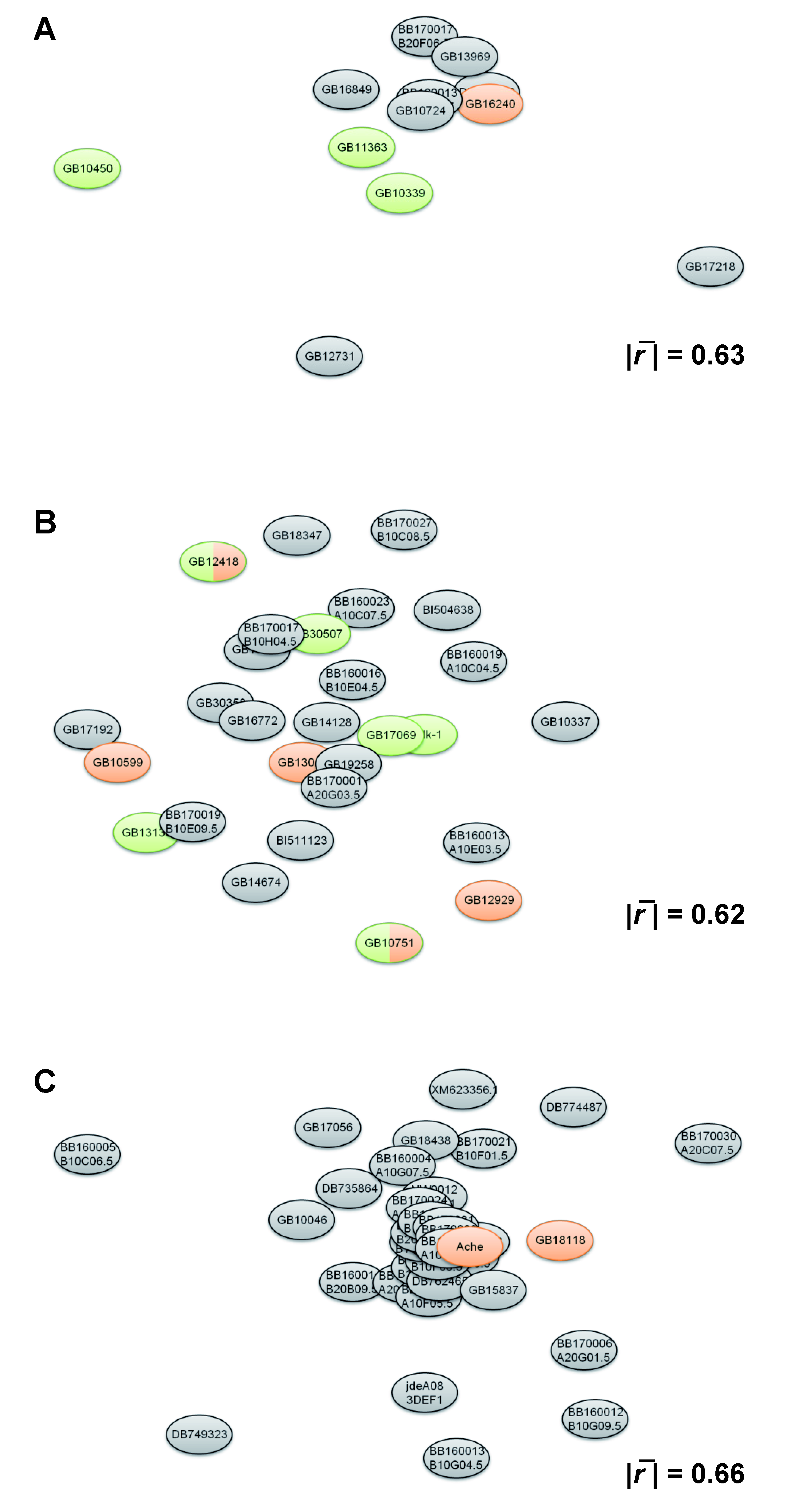

Supplement: Figure S4 — A network view of three highly co-regulated transcript sets after module formation. Correlated transcripts are shown in ovals with their gene names. The distances between genes in the plot correspond to their relative connectivity within the network, as calculated by a multiple scaling procedure. |r—| denotes the average correlation and connectivity of the entire transcript set. GO functional assignment: genes regulating developmental processes are shown in green, and genes controlling neuronal connectivity processes are marked in orange. (A) Module M3 consists of 12 transcripts (stress 0.06). (B) Module M4 includes 28 transcripts (stress 0.21). (C) Module M2 is comprised of 35 transcripts (stress 0.19). (TIFF) [file pone.0031653.s004.tiff]
