# Supplementary material for: Mixing of Honeybees with Different Genotypes Affects Individual Worker Behavior and Transcription of Genes in the Neuronal Substrate
Source: PLoS One. 2012 Feb 14;7(2):e31653. doi: 10.1371/journal.pone.0031653 (PMC3279409; doi:10.1371/journal.pone.0031653)
Supplement: Table S1 — The number of L bees that engaged in the uncapping task, but not also in the second hygienic task, removing, in mixed L/H and pure L bee groups (cumulated data). The observed numbers in mixed and pure L groups were compared with a χ2-test (df = 1; *P<0.025). (PDF) [file pone.0031653.s005.pdf]

Colony	# of L bees that engaged in		χ^2
	uncapping	uncapping and removing	
80%L/20%H	105	60	
100%L	51	52	
			5.2 *
