# Supplementary material for: Mixing of Honeybees with Different Genotypes Affects Individual Worker Behavior and Transcription of Genes in the Neuronal Substrate
Source: PLoS One. 2012 Feb 14;7(2):e31653. doi: 10.1371/journal.pone.0031653 (PMC3279409; doi:10.1371/journal.pone.0031653)
Supplement: Table S2 — The number of uncapped brood cells in mixed L/H bee groups (cumulative data from all cages). The expected numbers of uncapped cells (those that were treated) was estimated from the mean relative proportion of uncapped cells in pure L or H bee groups and the relative proportion of L and H bees in the mixed groups. The mean percentage of uncapped brood cells in H bee groups was 59% and 39% in L bee groups. The observed and expected numbers of uncapped cells in the different cages were compared with a χ2-test (df = 5; **P<0.0005). (PDF) [file pone.0031653.s006.pdf]

Colony genotypic mixing	# of uncapped cells		χ^2
	observed	expected	
80%L/20%H	32	16	
	17	15	
	21	15	
	15	13	
	17	12	
	6	12	
			23.9 **
